# Recurrent posterior circulation infarction caused by anomalous occipital bony process in a young patient

**DOI:** 10.1186/s12883-014-0252-6

**Published:** 2014-12-18

**Authors:** Seung-Hoon Song, Hong Gee Roh, Hahn Young Kim, Jin Woo Choi, Won-Jin Moon, Woo Jin Choe, Ileok Jung

**Affiliations:** Department of Neurology, Konkuk University School of Medicine, Neungdong-ro 120-1, Gwangjin-gu, Seoul, Republic of Korea; Department of Radiology, Konkuk University School of Medicine, Neungdong-ro 120-1, Gwangjin-gu, Seoul, Republic of Korea; Department of Neurosurgery, Konkuk University School of Medicine, Neungdong-ro 120-1, Gwangjin-gu, Seoul, Republic of Korea

**Keywords:** Recurrent strokes, Occipital bony process, Young age stroke, Endovascular treatment

## Abstract

**Background:**

Structural anomaly of the cervical spine or craniocervical junction has been reported as one of the rare causes of ischemic stroke. We report a case of a young patient with recurrent posterior circulation infarction that may have been associated with an anomalous occipital bony process compressing the vertebral artery.

**Case presentation:**

A 23-year-old man experienced recurrent posterior circulation infarction 5 times over a period of 5 years. He had no conventional vascular risk factors. Young age stroke work-up including thorough cardiac, intra- and extracranial vascular evaluation and laboratory tests for the hypercoagulable state or connective tissue disease yielded unremarkable results. An anomalous bony process from the occipital base compressing the left vertebral artery was observed on brain CT. All the recurrent strokes were explainable by the arterial thromboembolism originating from the compressed left vertebral artery. Therefore, the left vertebral artery compressed by the anomalous occipital bony process may have been the culprit behind the recurrent thromboembolic strokes in our patient. Intractable recurrent strokes even under optimal medical treatment led us to make a decision for the intervention. Instead of surgical removal of the anomalous occipital bony process, the left vertebral artery was occluded permanently by endovascular coiling after confirming that this would cause no neurological deficits or flow disturbance in the posterior circulation. There was no recurrence of stroke for 2 years after permanent occlusion of the left vertebral artery.

**Conclusion:**

Arterial thromboembolism originating from the left vertebral artery compressed by the anomalous occipital bony process is a rare but not to be overlooked cause of posterior circulation infarction. When intractable to medical treatment, endovascular occlusion of the vertebral artery without flow disturbance to the posterior circulation may be a useful treatment option when surgical removal is not feasible.

## Background

Cerebral infarction in young people is rare. Infarction at a young age may have distinct causes such as hypercoagulability, connective tissue disease, cerebral or cervical arterial dissection, or cardiac problems [1-3]. Particularly, recurrent cerebral infarction indicates a higher possibility of the presence of unusual underlying causes. Structural anomaly of the cervical spine or craniocervical junction has been reported as one such unusual cause of infarction [4,5]. We report the case of a young patient with recurrent posterior circulation infarction that may have been associated with an anomalous occipital bony process compressing the vertebral artery.

## Case presentation

A 23-year-old man had a history of recurrent cerebral infarction localized to the vascular territory of posterior circulation. He had no conventional vascular risk factors for stroke. Laboratory findings including lipid profile, D-dimer level, and autoimmune antibody tests were unremarkable. In addition, cardioembolism was excluded after a detailed cardiological evaluation including electrocardiography, echocardiography, 24-hr Holter monitoring, and cardiac CT.

**Figure 1:**
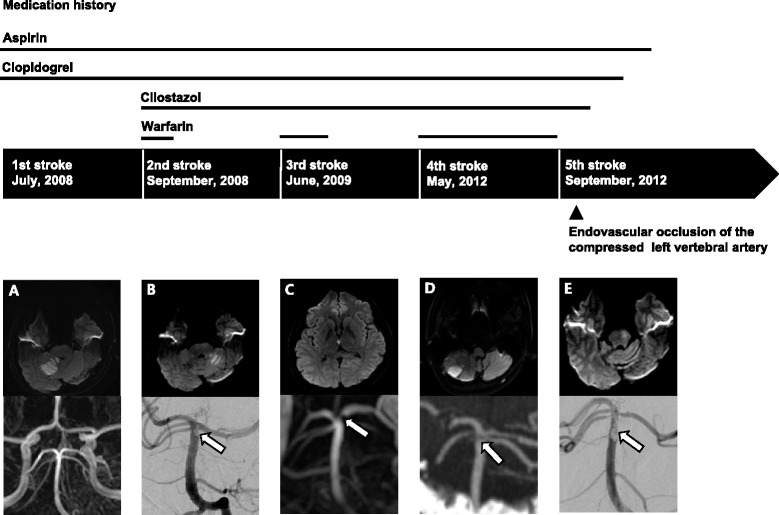
**Medication history and findings of diffusion-weighted MRI and angiography of five recurrent strokes.** (**A:** first stroke; **B:** second stroke; **C:** third stroke; **D:** fourth stroke; **E:** fifth stroke). Recurrent thromboemboli were observed in the distal basilar artery (arrows). Finally, the left vertebral artery was permanently occluded with detachable coils after the fifth stroke.

**Figure 2:**
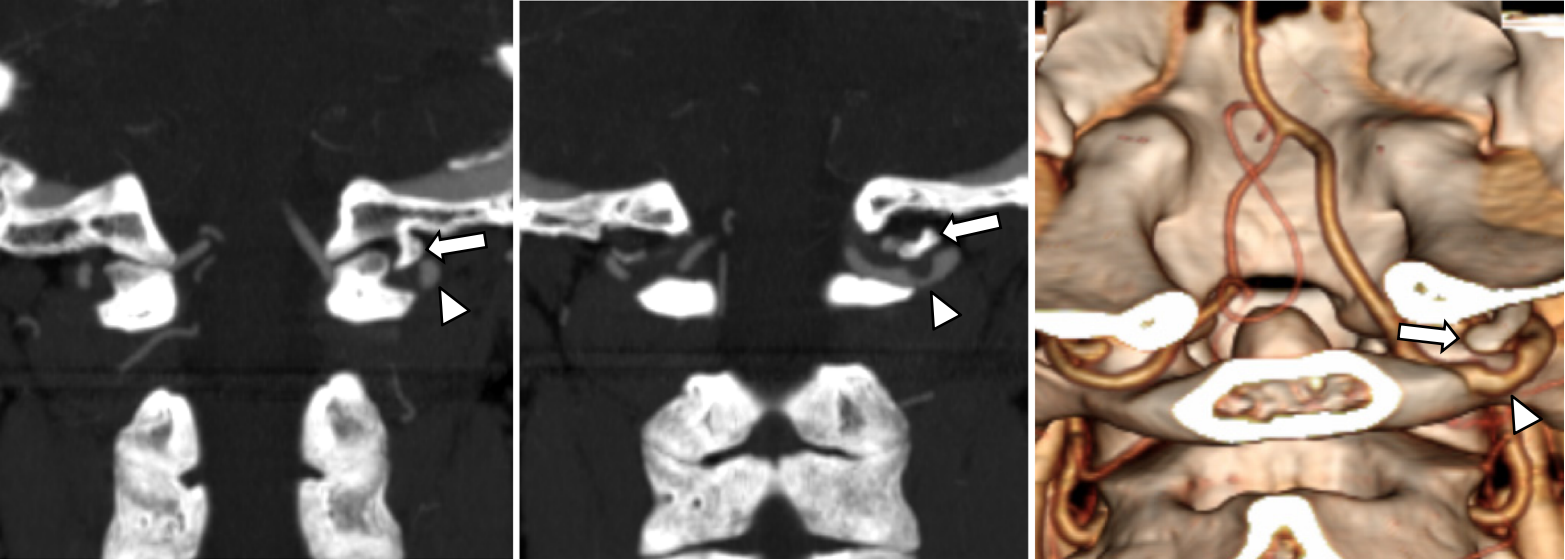
**Anomalous occipital bony process compressing the vertebral artery.** CT angiography showed the left vertebral artery (arrowheads) compressed by the anomalous occipital bony process (arrows).

In his first stroke, he experienced dizziness of sudden onset (first stroke, July 2008). Brain MRI showed acute infarction in the vascular territory of the right superior cerebellar artery (Figure 1A). He started taking aspirin (100 mg), clopidogrel (75 mg), and atorvastatin (10 mg). Interestingly, a small hook-shaped anomalous occipital bony process from the left occipital base compressing the left vertebral artery was observed (Figure 2). Although we could not exclude the possibility of arterial thromboembolism originating from the compressed left vertebral artery, we preferred medical treatment instead of invasive intervention.

Two months later, he experienced dizziness again (second stroke, September 2008). Brain MRI showed acute infarction in the left cerebellum and paramedian pons with thromboembolus at the distal basilar artery (Figure 1B). Then, cilostazol (200 mg) and warfarin were added to his regimen. One month later, warfarin was withdrawn for the concern of bleeding in long-term anticoagulation. However, triple antiplatelet therapy including aspirin, clopidogrel, and cilostazol was continued.

Nine months later, he experienced left arm weakness (third stroke, June 2009). Brain MRI revealed acute infarction in the right medial thalamus and paravermian cerebellum along with thromboembolus at the distal basilar artery (Figure 1C). Warfarin was added again to triple antiplatelet therapy. He recovered without neurological deficit. Ten months later, warfarin was withdrawn again for the concern of bleeding in long-term anticoagulation. Three years later, he experienced dysarthria and ataxia (fourth stroke, May 2012). Acute infarction was found in the vascular territory of the left posterior inferior cerebellar artery. CT angiography revealed thromboembolus at the distal basilar artery again (Figure 1D).

**Figure 3:**
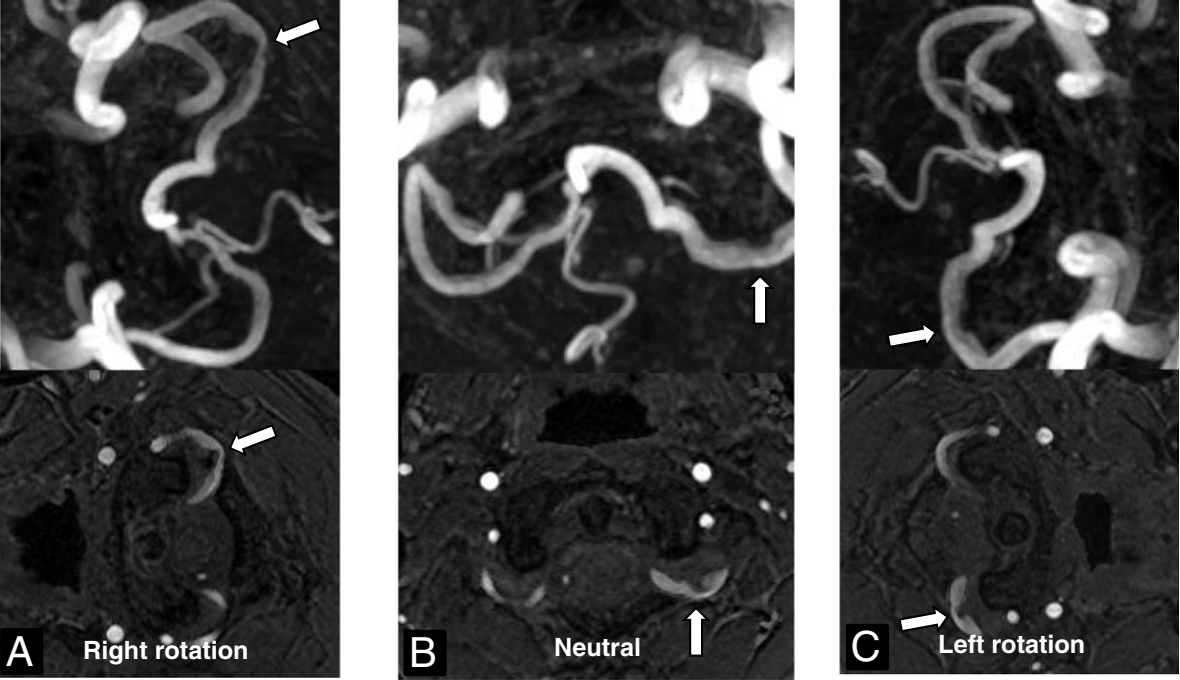
**Dynamic time-of-flight MR angiography showed that left vertebral artery stenosis (arrows) was slightly aggravated when the head was turned to the right side (A: right head rotation, B: neutral position, C: left head rotation).**

**Figure 4:**
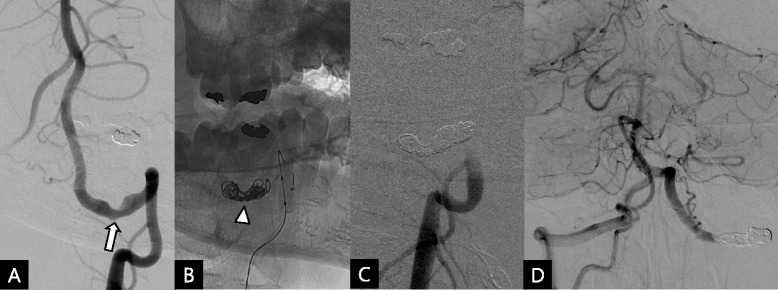
**Procedure of endovascular occlusion of the left vertebral artery. A**. Left vertebral artery angiography showed stenosis (arrow). **B**. The first detachable coil (arrowhead) was placed across the stenotic segment with proximal flow control with microballoon catheter. **C**. The left vertebral artery was completely occluded after the coil embolization. **D**. The right vertebral artery sufficiently took over the entire posterior circulation territory perfusion. The left vertebral artery is opacified retrogradely.

To examine the causal relation between recurrent arterial thromboembolism and compression of the left vertebral artery, dynamic time-of-flight MR angiography was performed in 3 positions: neutral, right-, or left-sided head rotation. Stenosis of the left vertebral artery was observed in the neutral position and was slightly aggravated when the head was turned to the right side (Figure 3). There was no remarkable finding in the repeated complete stroke work-ups except for stenosis of the left vertebral artery. Therefore, we were convinced about the causal relationship between recurrent arterial thromboembolism and stenosis of the left vertebral artery compressed by the anomalous occipital bony process. Because the third and fourth strokes recurred when anticoagulation therapy was discontinued, we added warfarin again in addition to the triple antiplatelet therapy. However, unfortunately, the fifth stroke presenting right-sided weakness and facial palsy occurred under adequate anticoagulation and triple antiplatelet therapy (fifth stroke, September 2012). Acute left paramedian pontine infarction and irregular thromboembolus at the distal basilar artery were observed (Figure 1E). Emergency intra-arterial suction thrombectomy was performed and the basilar artery was completely recanalized. Conventional cerebral angiography revealed compression of the left vertebral artery even in the neutral position and was slightly aggravated when the head was rotated to the right side. However, there was no definite flow disturbance according to head rotation. Because of the recurrent infarctions under optimal medical treatment including anticoagulation and triple antiplatelet therapy, we decided to perform endovascular occlusion of the left vertebral artery by coiling. He tolerated the internal trapping procedure following balloon occlusion test without any neurological complication (Figure 4). Triple antiplatelet therapy was continued for 5 months and tapered one by one. Currently, there has been no recurrence of stroke for 2 years after permanent occlusion of the left vertebral artery.

## Discussion

### Stroke mechanism

In the previous studies, the stroke mechanism of patients with infarction aged below 50 years was reported [1-3]. According to the Trial of Org 10172 in Acute Stroke Treatment (TOAST) classification of stroke mechanism, the proportion of other determined etiology in young–age stroke is rather high: large-artery atherosclerosis, 11%, cardioembolism, 24%, small-artery occlusion, 8%, stroke of other determined etiology, 27%, and stroke of undetermined etiology, 29% [3]. Vertebral arterial injuries associated with structural osseous anomaly, one of other determined etiology, have been reported [4,5].

Anomalous occipital bony process may be a byproduct of fusion abnormalities [6-8]. The incidence of anomaly of atlanto-occipital fusion that could cause compression of the vertebral artery varies between 0.5% and 1.0% [6,7]. The symptoms may be headache, neck pain, numbness, and weakness in the limbs, or cranial nerve dysfunction including tinnitus, visual disturbance, dysphagia, or dysarthria [8]. The vertebral artery may be compressed mechanically during head rotation by adjacent bony structures and dynamic disturbance of blood flow can occur [5,9]. Bow hunter’s syndrome is defined as symptomatic vertebrobasilar insufficiency caused by transient mechanical occlusion of the vertebral artery during head rotation [10]. Meanwhile, vertebral artery injury attributable to the recurrent compression by the anomalous occipital bony process causing thrombosis may give rise to infarction [4]. In our patient, the anomalous occipital bony process had been compressing the vertebral artery even in the neutral position and no significant flow disturbance was induced by head rotation. Therefore, the recurrent thromboembolism in our patient might have originated from the endothelial injury of the compressed vertebral artery rather than from direct mechanical compression by head rotation. The stroke mechanism in our patient may be different from that in typical bow hunter’s syndrome. A focal stenotic signal between the occipital bony process and vertebral arterial lumen may suggest the presence of endothelial or perivascular inflammatory tissue or the occurrence of subintimal dissection caused by repetitive mechanical compression. Therefore, we were concerned about the possibility that residual organized stenosis even after the surgical removal of the anomalous occipital bony process may work as a persistent embolic source. No further stroke after permanent occlusion of the left vertebral artery may provide evidence that recurrent arterial thromboembolism originating from the compressed left vertebral artery might be the cause of recurrent posterior circulation infarction in our patient.

### Treatment plan

After the second and third stroke, we added warfarin to the triple antiplatelet therapy. However, when warfarin was discontinued, the stroke recurred (third and fourth strokes). The fifth stroke occurred even under adequate anticoagulation and triple antiplatelet therapy. Therefore, we considered the following treatment options: 1) anticoagulation with higher international normalized ratio (INR 2.5-3.5) and triple antiplatelet therapy, 2) surgical resection of the anomalous occipital bony process, or 3) endovascular occlusion of the vertebral artery.

Life-long anticoagulation with higher INR and triple antiplatelet therapy in a young patient was considered risky owing to the possibility of bleeding complications in long-term treatment. Cases of young patients with recurrent posterior circulation infarction associated with the occipital bony process have been reported recently [4,5]. The authors suggested that the bony protuberance possibly caused thrombi formation, and surgical removal was performed successfully as a permanent treatment [4,5]. Considering the relatively smaller contralateral vertebral artery in our patient, surgical removal of the anomalous occipital bony process, saving both the vertebral arteries, might have been the best treatment option. However, in addition to the risk of surgical complications in cervical spine surgery (4-8%) [11,12], residual persistent organized stenosis of the vertebral artery even after the surgical removal of anomalous occipital bony process may work as a future recurrent embolic source. Hence, we chose endovascular treatment instead of surgical removal. Because the contralateral vertebral artery was smaller, the left vertebral artery was occluded after confirming the absence of neurological deficits or flow disturbance in the posterior circulation when it was temporarily occluded during the endovascular procedure (Figure 4). Although there was no recurrence of stroke until 2 years after the permanent occlusion of the left vertebral artery, the long-term outcomes need to be followed to confirm that this procedure is safe as a permanent treatment.

## Conclusion

Arterial thromboembolism originating from the left vertebral artery compressed by the anomalous occipital bony process is a rare but not to be overlooked cause of posterior circulation infarction. When intractable to medical treatment, endovascular occlusion of the vertebral artery without flow disturbance of the posterior circulation may be a useful option when surgical removal is not feasible.

## Consent

We obtained written informed consent from the patient for publication of this case report and any accompanying images.
